# Characterisation of a 4A QTL for Metribuzin Resistance in Wheat by Developing Near-Isogenic Lines

**DOI:** 10.3390/plants10091856

**Published:** 2021-09-07

**Authors:** Rudra Bhattarai, Hui Liu, Kadambot H. M. Siddique, Guijun Yan

**Affiliations:** 1 UWA School of Agriculture and Environment, The University of Western Australia, 35 Stirling Highway, Crawley, WA 6009, Australia; rudra.bhattarai@research.uwa.edu.au (R.B.); kadambot.siddique@uwa.edu.au (K.H.M.S.); 2 The UWA Institute of Agriculture, The University of Western Australia, 35 Stirling Highway, Crawley, WA 6009, Australia

**Keywords:** metribuzin resistance, near-isogenic lines, quantitative trait locus, wheat, leaf chlorophyll

## Abstract

Wheat (*Triticum aestivum* L.) production is constantly affected by weeds in the farming system. Chemical-based weed management is widely practiced; broad-spectrum herbicides such as metribuzin have been successfully used to control weeds in Australia and elsewhere of the world. Breeding metribuzin-resistant wheat through genetic improvement is needed for effective control of weeds. Quantitative trait loci (QTLs) mapping efforts identified a major QTL on wheat chromosome 4A, explaining up to 20% of the phenotypic variance for metribuzin resistance. The quantitative nature of inheritance of this QTL signifies the importance of near-isogenic lines (NILs), which can convert a quantitative trait into a Mendelian factor for better resolution of the QTL. In the current study, NILs were developed using a heterogeneous inbred family method combined with a fast generation-cycling system in a population of Chuan Mai 25 (resistant) and Ritchie (susceptible). Seven pairs of NILs targeting the 4A QTL for metribuzin resistance were confirmed with a molecular marker and phenotyping. The resistant allele from the resistant parent increased metribuzin resistance by 47–86% (average 63%) compared with the susceptible allele from the susceptible parent. Segregation analysis in the NIL pairs for thousand grain weight (TGW) (g), biomass per plant (kg), tillers per plant, plant height (cm), yield per plant, and powdery mildew visual score (0–9) indicated that these traits were linked with metribuzin resistance. Similarly, TGW was observed to co-segregate with metribuzin resistance in most confirmed NILs, signifying that the two traits are controlled by closely linked genes. The most contrasting NILs can be further characterised by transcriptomic and proteomic analyses to identify the candidate genes responsible for metribuzin resistance.

## 1. Introduction

Wheat is a major food source in the world. Weeds constantly affect wheat production, competing for moisture, nutrient, and light resources, resulting in plant height reduction, nutrient starvation, and wilting [1]. Weed infestation may reduce wheat yields, sometimes by 50% [2]. Every year, weeds cost Australian agriculture an estimated $2.5 billion to $4.5 billion per annum [3]. Therefore, controlling weeds is a prerequisite for safe agricultural production. Controlling weeds with herbicides is one of the most common and effective weed control measures [4]. Metribuzin is a broad-spectrum herbicide used extensively in dryland farming systems in Australia [5]. It is a triazine herbicide that interferes with photosystem II (PSII) electron transport in plant chloroplasts [6]. Being a broad-spectrum herbicide, metribuzin can also harm valuable crops, such as wheat in the field. The development of metribuzin resistance in wheat could occur through wheat lethal dose identification, screening wheat cultivars for resistance to metribuzin, and introgression of a resistance source. To introduce such resistance genes, overall gene characterisation is essential.

Genetic loci responsible for metribuzin resistance have been reported in numerous plant species. Metribuzin-resistance genetic studies in wheat identified sources of resistance [7], quantitative trait loci (QTLs), and putative candidate genes [7]. A QTL mapping study identified three QTLs, one each on chromosomes 1A, 4A, and 2D [8]. Another high-density genetic linkage map of a Chuan Mai 25/Ritchie population identified seven QTLs on chromosomes 2A, 2D, 3A, 3B, 4A, 5A, and 6A [7]. The 4A QTL was identified in both mapping populations—Synthetic W7984/Opata 85 [8] and Chuan Mai 25/Ritchie [7] and the two separate QTL mapping studies. The 4A QTL contribute up to 20% of the phenotypic variance for metribuzin resistance. So far, no effort has been undertaken to further understand the genetics of the traits for metribuzin resistance and to characterise this important QTL.

Successful development of NILs using QTL mapping information can convert a complex quantitative trait into a Mendelian factor [9] to accurately localise the genes controlling the trait, enhancing the mapping resolution. Contrasting isolines are assumed to be similar in genetic backgrounds so that the trait differences between them can be attributed to the targeted gene or locus and nearby genes, facilitating the discovery of the effects of this locus or region on other traits [10]. Similarly, NILs are valuable for accurate trait localisation and ideal for studying phenotypic effects attributable to a particular trait [11].

Many factors limit the practical use of QTL markers directly in breeding programs. Firstly, large genomic intervals associated with the QTL region make them less suitable for target trait selection as there may be recombination between the markers and genes [12]. Secondly, accurate phenotyping of the desired trait might be affected by the segregation of QTLs in the targeted region [13]. However, a QTL marker can be used to develop NILs [14]. This study uses markers close to the QTL to develop a series of NILs. Single markers have been used to develop NILs successfully in barley and wheat [15,16,17]. Putative NILs were developed through repeated selfing using the heterogeneous inbred family (HIF) method [18] and a fast generation-cycling system (FGCS) [19]. FGCS helped to speed up the generation advancement process without losing any recombination benefit and significantly increased population development efficiency [19,20].

Here, we report the development, confirmation, and genotypic and phenotypic characterisation of NILs targeting the major 4A QTL, responsible for metribuzin resistance in wheat.

## 2. Results

### 2.1. Development of Near-Isogenic Lines

**Figure 1:**
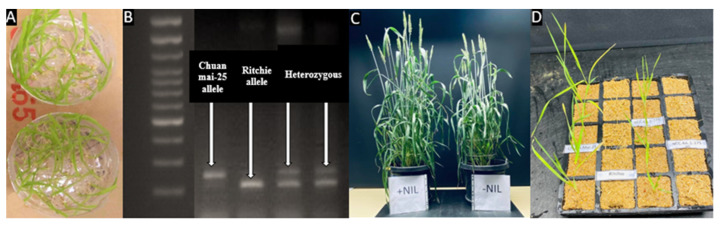
Procedures involved in NIL development. (**A**) Seedlings raised using a fast generation-cycling system (FGCS): the extracted embryos were germinated, and the plantlets grown in tissue culture media on Petri dishes. (**B**) A molecular marker was used to select the homozygous resistant (Chuan Mai 25 allele) and homozygous susceptible (Ritchie allele) lines in the F_8_ generation while the heterozygous types were selected up to the F_7_ generation. (**C**) One NIL pair (one line with Chuan Mai 25 allele ‘+’and another line with Ritchie allele ‘−’) in the F_8_ generation differed in growth. (**D**) Columns from left to right: resistant parent Chuan Mai 25 seedlings, susceptible parent Ritchie seedlings (all dead), resistant isolines of a NIL pair ‘17′, and susceptible isolines of pair ‘17′ (all dead).

**Figure 2:**
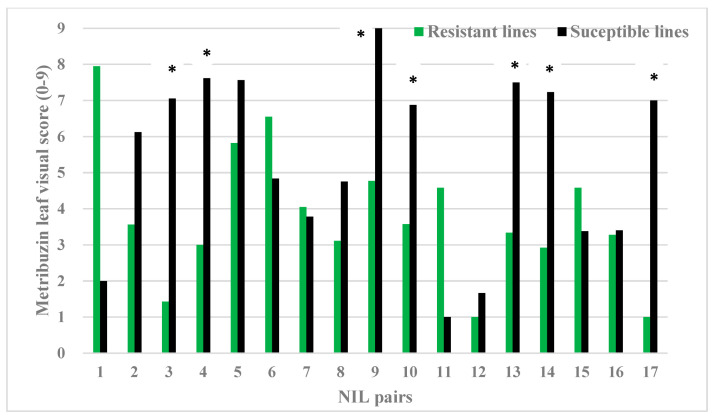
Seventeen pairs of putative near-isogenic lines (NILs) derived from F_2_ single-seed descent progenies conferring metribuzin resistance in the Chuan Mai 25 × Ritchie population were assessed with the metribuzin leaf visual score (0–9: score 0 is the most resistant and score 9 means most susceptible), which was taken on the 12th day of metribuzin application. Lines with the resistant molecular marker are green, and lines with the susceptible molecular marker are black. Confirmed NILs are marked with *.

Seventeen heterozygous lines were identified in the F_7_ generation using the HIF method (Table S1). Two contrasting lines, each homozygous to the molecular marker alleles, were selected from the progeny of each F_7_ heterozygous plant. At F_8_, 17 putative NIL pairs were obtained and used for the metribuzin assessment. Of the 17 pairs, seven were confirmed NIL pairs against metribuzin, and others were confirmed as recombination types (Figure 1A–D and Figure 2).

### 2.2. Evaluation of NIL Pairs Based on Visual Scoring (0–9) and SPAD Measurement after Metribuzin Treatment

Leaf browning in susceptible isolines started from 9 days after treatment but was not clearly differentiated until 12 days after treatment. Metribuzin-based phenotyping and statistical analysis revealed significant (*p* < 0.05) to highly significant (*p* < 0.01) differences in the leaf visual score (0–9: score 0 is the most resistant and score 9 means most susceptible) and leaf SPAD chlorophyll content between isolines for each confirmed NIL pair (Table 1). Significant differences occurred between the isolines of pairs 3, 4, 10, 14, and 17 (*p* ≤ 0.01) and pairs 9 and 13 (*p* ≤ 0.05) for the metribuzin leaf visual score (0–9) using a non-parametric *U* test (Table 1). Resistant isolines had lower leaf visual scores with dark green leaves (Figure 1D) than susceptible isolines with dead leaves. The resistant allele from the resistant parent increased metribuzin resistance by 47–86% (average 63%) compared with the susceptible allele from the susceptible parent, based on visual scoring (Table 1).

Significant differences occurred between the isolines of pairs 3, 10, 13, and 17 (*p* ≤ 0.01) and pairs 4, 9, and 14 (*p* ≤ 0.05) for SPAD leaf chlorophyll content (Table 1). Resistant isolines of each NIL pair had a higher leaf SPAD chlorophyll content than the susceptible isolines. The SPAD measurement outcome generally corresponded with the leaf visual score assessments (Table 1). The resistant allele from the resistant parent increased metribuzin resistance by 20–48% (average 33%) compared with the susceptible allele from the susceptible parent, based on leaf SPAD chlorophyll content (Table 1).

### 2.3. Evaluation of NIL Pairs for Morphological Traits

Morphological traits were further investigated in each of the seven NIL pairs. Some NIL pairs significantly differed in morphological traits. Highly significant differences occurred between the isolines of pairs 9 and 14 for tillers per plant (*p* ≤ 0.01); pairs 3, 4, 10, 13, and 17 for TGW (g) (*p* ≤ 0.01); pairs 10, 13, and 14 for biomass (kg) per plant (*p* ≤ 0.01); and pairs 3, 14, and 17 for plant height (cm) (*p* ≤ 0.01) (Table 2). Similarly, significant differences occurred between the isolines of pair 13 for tillers per plant (*p* ≤ 0.05), pair 9 for TGW (g) (*p* ≤ 0.05) and biomass (kg) per plant (*p* ≤ 0.05), and pair 14 for TGW (g) (*p* ≤ 0.05). Highly significant differences occurred between the isolines of pair 4 for yield (g) per plant (*p* ≤ 0.01). In turn, pairs 9, 10, and 13 were found significantly different (*p* ≤ 0.05) in per plant grain yield. Isolines of pairs 4, 9, and 10 were found highly significantly different for the powdery mildew (PM) visual score (0–9) (Table 2).

## 3. Discussion

This study confirmed seven pairs of NILs targeting the 4A locus [8,21] resistance in bread wheat. A significant difference in leaf SPAD chlorophyll content and leaf visual score (0–9) between the isolines of the NIL pairs against metribuzin confirmed the significance of targeted QTL for metribuzin resistance. The confirmed NIL pairs were also analysed for other morphological traits to study the traits that might link to the metribuzin resistance locus; for example, the leaf chlorophyll content may be controlled by photosystem-related genes, which can contribute to photosynthate partitioning for yield-related traits in crops [22]. The PSII component of leaf chloroplasts, the major component of leaf chlorophyll content, is affected by metribuzin [23]. Several studies have highlighted the importance of metribuzin-resistant QTLs in wheat for maintaining photosynthetic capacity [21,24,25]. In this study, the developed NILs with a Chuan Mai 25 allelic background had a higher leaf SPAD chlorophyll content than those with a Ritchie allelic background. This is because the marker used for developing the NILs, Xbarc343, is linked to the photosystem-related genes present in chloroplasts [8]. In the same study, the leaf SPAD chlorophyll content and metribuzin leaf visual score were reliable indicators for evaluating metribuzin resistance [8]. The usefulness of these two measurements in determining metribuzin resistance can be further improved through the successful development and confirmation of NILs.

We recommend using the leaf visual score (0–9) and SPAD chlorophyll content for morphological evaluation of metribuzin resistance, as they are easy, rapid, and inexpensive to measure. In this study, the HIF method was used to generate the NILs. The confirmed NILs from the genotype–phenotype (metribuzin-based phenotyping) were enhanced through characterisation by determining the association strength of the metribuzin resistance trait with different morphological traits. NIL development traditionally takes time, with only two or three generations achieved per year, and is expensive. However, a recently developed FGCS offers up to eight generations per year. By using young embryos germinated on media, this procedure dramatically shortens the life cycle due to the short post-anthesis stage and no after-ripening stage needed to proceed to next generation. However, FGCS requires aseptic conditions and handling of young seeds to transfer germinated seedlings into the soil for growth and development. Even so, FGCS, assisted by DNA marker-assisted selection, allowed us to develop NILs quickly in this study.

To select the heterozygous and homozygous lines, we used a single marker from the targeted 4A QTL to minimise the genetic dragging of the undesirable chromosomal fragments that could be selected unintentionally when using multiple flanking markers [26]. Undesirable chromosomal dragging is even possible from selecting using a single marker, though the probability is less. Additionally, if the marker is too far away from the QTL, the marker–trait association can be lost [27], such that the marker may not represent the targeted trait. As a result, only seven NILs were confirmed as true NILs.

**Figure 3:**
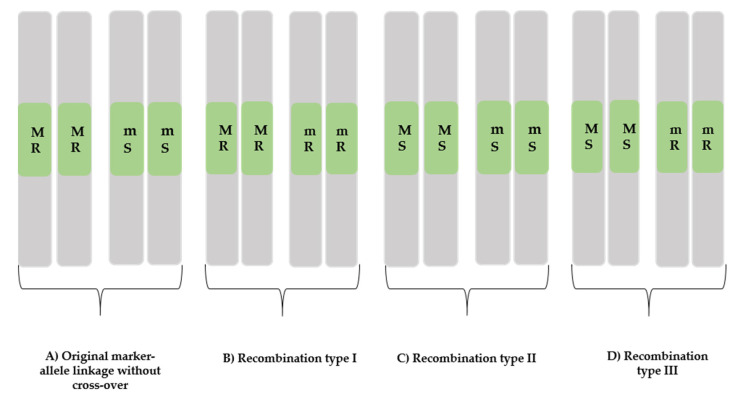
Four types of outcomes were observed in the heterogeneous inbred lines during near-isogenic line (NIL) development, when non-recombination or recombination occurred. The above-mentioned NIL pairs were obtained from selfing F_7_ heterozygous lines derived from F_2_ single-seed descent progenies. R in green boxes indicates the gene allele responsible for metribuzin resistance, and S in green boxes indicates the gene allele for metribuzin susceptibility. M in green boxes represents the resistant molecular marker from the resistant parent Chuan Mai 25, and m in green boxes represents the susceptible molecular marker from the susceptible parent Ritchie. (**A**) Marker–allele linkage type. (**B**–**D**) Recombination types.

**Figure 4:**
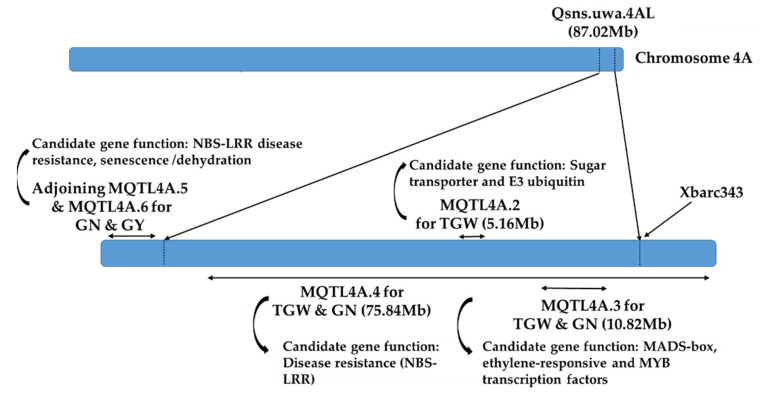
Conceptual framework of the meta QTLs (MQTLs) [32] reported in the targeted 4A locus. MQTL4A.2, MQTL4A.3 and MQTL4A.4 were found overlapping with the 4A locus, and MQTL4A.5 and MQTL4A.6 were adjacent to it. The numbers in the brackets indicate the physical positions of the QTL.

The progenies of the heterozygotes with the marker and QTL crossover during meiosis yielded four outcomes (Figure 3). The A-type NIL pairs were contrasting for the measured traits with a marker–gene linkage: the resistance marker allele was linked to the metribuzin-resistant phenotype, and the susceptible marker allele was linked to the metribuzin-susceptible phenotype. In addition, one isoline of each A-type NIL pair (3, 4, 9, 10, 13, 14, and 17) (Table 1) was resistance against metribuzin, and the other was susceptible (Figure 3). Though, they have variabilities in response to metribuzin (Table 1), as they were the descendent progenies of different single seeds from F_2_. This is why the obtained NIL pairs were found diverse in genetic backgrounds during evaluation, which was confirmed from the segregation of morphological traits in the different NILs. Targeted trait metribuzin resistance in the current research was mostly co-segregated with TGW (Table 2) in most confirmed NIL pairs (Table 1). Similarly, plant biomass per plant in pairs 9, 10, 13, and 14; plant height in pairs 3, 14, and 17; tillers per plant in pairs 9, 13, and 14; and yield per plant in pairs 4, 9, 10, and 13 were segregated differently (Table 2) in different confirmed NILs, indicating that they are metribuzin-resistance-linked traits influencing the metribuzin resistance. This finding is consistent with [28] a QTL mapping study in which qGY4A for grain yield and qChlb4A for chlorophyll content was found to co-segregate. These two QTLs intervals’ physical positions were found to overlap with the interval of the current 4A QTL [8]. A recent study from [29] also reported the same QTL region controlling other morphological traits, such as thousand-grain weight, grain number (GN), grain yield (GY), and tolerance to abiotic and biotic stresses, with the candidate genes including functions related to NBS-LRR disease resistance in wheat, etc. (Figure 4). In the current study, powdery mildew (PM) was found highly significantly different between isolines of the confirmed NIL pairs (4, 9, and 10) (Table 2), suggesting that they were linked traits probably controlled by independent genes within the locus. Despite having variabilities for metribuzin resistance/susceptibility (Table 1) in the developed NILs, the linkage of the metribuzin-resistance trait and the flanking marker Xbarc343 was proved by the successful development of the contrasting NILs, confirming the usefulness of the QTL to breed metribuzin resistance in wheat. More interestingly, all the confirmed NIL pairs except for pair 14 obtained in this study differed significantly to highly significantly for TGW, indicating that both traits are likely to be governed from closely linked genes (Table 2). Seed related traits responsible for herbicides resistance has also been reported in several species [30]. A recent study also reported that seed size in wheat was determined through the mechanism related to seed integuments proliferation [31]. The physiology behind such mechanism has not been properly studied, but it has been reported that higher proliferation of integuments increased the cells number in the seed coat, resulting in seed enlargement [32]. Such increased cell numbers in seed coats could act as a barrier against metribuzin through obstructing the imbibition mechanism or cause a delay to reach metribuzin to the targeted tissue. Seed mass was positively associated with better seedling survival; larger seeds generally result in larger seedlings [33]. Larger seedlings generally have a greater ability to escape from various hazards, including drugs and chemicals [34,35]. The linkage between seed size and metribuzin resistance at this locus needs further investigation. Therefore, the detailed molecular characterisation of the confirmed NILs through expression profiling or fine mapping is necessary to identify the functional gene(s) for metribuzin resistance and their linkage to other traits.

Isolines of the B and C types (Figure 3) lacked differences between the NIL pairs in the majority of morphological traits measured, indicating the possibility of a recombination happening in the QTL region. A fine mapping with a higher resolution genetic map and closely linked markers in the QTL region would reduce such a recombination issue [36]. The D type (NIL pairs 1 and 11) was also a recombination type, with contrasting performance against metribuzin. The isolines with the resistance allele showed the susceptible phenotype, and the isolines with the susceptibility allele showed resistance against metribuzin. This suggests that double recombination happened, resulting in the phenotyping acting like the metribuzin-resistance gene switched.

## 4. Materials and Methods

### 4.1. Plant Material

A cross-population of Chuan Mai 25 (metribuzin-resistant parent) and Ritchie (metribuzin-susceptible parent) [7] was used to develop the NILs for this study.

### 4.2. Development of NILs

**Figure 5:**
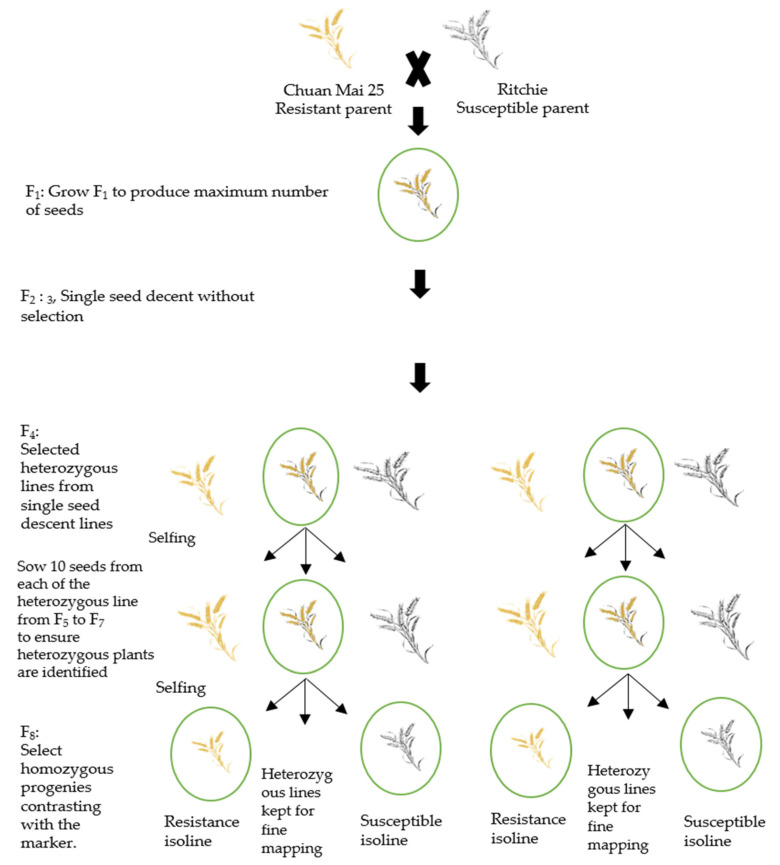
Process for developing near-isogenic lines (NILs). The heterogeneous inbred family method (Tuinstra et al. 1997) was used to develop the NIL pairs. Molecular markers were used to select heterozygous lines from F_4_ to F_7_. Putative near-isogeneic line pairs were selected at F_8_ from each of the F_7_ heterozygous lines.

Chuan Mai 25 and Ritchie were crossed to produce F_1_ seeds. The single-seed descent (SSD) method was used to generate the populations—the lines were sown individually and harvested separately for each generation. F_4_ lines were produced using the SSD method from F_2_ to F_4_ without selection. A flanked DNA marker from the QTL interval was used to select the heterozygous lines in each generation from F_4_ onwards. At least ten seeds from each identified heterozygous plant were used from F_5_ to F_7_ to ensure suitable heterozygous lines for inclusion in the selection. For every generation from F_4_ to F_7_, immature seeds (10 to 15 days after anthesis) were collected, dissected, and cultured based on FGCS [20]. A selected F_7_ heterozygous plant was selfed to produce a pair of F_8_ generation lines that were homozygous at the target locus but contrasting for the marker alleles to be used as a putative NIL pair (Figure 5), assuming 99.21% identical genetic background except for the targeted marker locus. Isoline pairs were, therefore, distinct at the marker allele, either with the resistance allele from Chuan Mai 25 (R lines) or the susceptible allele from Ritchie (S lines) (Figure 1B). Selfed F_8_ putative NIL seeds were used for the metribuzin assessment and agro-morphological evaluation to confirm metribuzin resistance and to identify the potential traits interacting with metribuzin resistance, respectively.

### 4.3. Molecular Marker Analysis

Genomic DNA was isolated from the leaves of three-week-old seedlings using a modified CTAB method [37]. A metribuzin-resistant QTL flanking SSR (simple sequence repeat) marker, Xbarc343 [8], was used to identify the heterozygous/homozygous lines from the segregating population. Primer sequences were forward GGCCTAATTACAAGTCCAAAAG, and reverse GCTCAAAGTAAAGTTCACGAATAT, respectively. PCR reactions were performed in 15 µL containing 100–250 ng template DNA (1 µL), 5 µM each primer (forward and reverse) (0.3 µL each), 1.5 µL of 10X PCR buffer (with MgCl_2_), 10 mM dNTPs mix (2.4 µL), and 1 unit of *Taq* DNA polymerase (Sigma-Aldrich, Australia) mixed in 8.78 µL of PCR-grade water, with the following program: denatured at 94 °C for 5 min, 35 cycles of denaturation at 94 °C for 30 s, annealing at 52 °C for 45 s, and extension at 72 °C for 45 s, with a final extension at 72 °C for 7 min. The amplified fragments were electrophoresed on 1.5% agarose gel, stained with ethidium bromide, and visualized under UV light.

### 4.4. Fast Generation-Cycling System

We used FGCS, an embryo-culture based technique where immature seeds are harvested from wheat at the GS70 to GS80 growth scale [38]. The collected young grains were sterilised and embryos extracted. Embryos were grown under aseptic condition and cultured in a growth medium described by [19]. One-week-old young seedlings (Figure 1A) were transferred into the soil in 20-cell Kwikpot trays (Rite-Gro Kwikpots, GardenCity Plastics) (25 cm × 25 cm × 6 cm). About two weeks of transferring, the plantlets were ready for DNA extraction to identify the heterozygous lines for next-generation advancement.

### 4.5. Metribuzin Assessment of Putative NILs

Kwikpot trays filled with homogenous river sand (Figure 1D) were watered to 100% field capacity. A NIL pair of 20 seeds were placed in 20 uniform cells seedling trays (five rows by four columns). There were ten seeds per isoline in a tray, where five seeds from each isoline were placed in a column alternatively with the opposite isoline. Seventeen NIL pairs were placed in seventeen such trays for the treatment and control. The herbicide recommended dose (200 g ai ha^−1^) for controlling weeds in wheat fields in Western Australia (https://www.titanag.com.au/Products/Metribuzin_750_WG_PM.pdf, accessed on 27 February 2021) was sprayed via a twin flat-fan nozzle perpendicular to the direction of sowing in two passes at pressure of 200 kPa in a spray chamber calibrated to deliver 106 L water ha^−1^. Control pots were sprayed with deionized (DI) water. The trays were maintained in the glasshouse set at 25/15 °C day/night with light intensity ranging from 400 to 600 mmol m^−2^ s^−1^ and watered every 48 h.

### 4.6. Phenotyping

The chlorophyll content of the NIL pairs was measured using a portable Minolta SPAD-502 chlorophyll meter (Spectrum Technologies, Inc., Plainfield, IL, USA) on the 12th day of metribuzin application. On the 12th day of application, isolines of most NIL pairs had also shown visual difference between treatments: green leaf retention related to higher leaf SPAD chlorophyll content versus dead or nearly dead leaves related to lower leaf SPAD chlorophyll content, indicating resistance and susceptibility, respectively (Figure 1D). Among the seventeen putative NIL pairs, the most contrasting pairs were selected based on differences in the leaf SPAD chlorophyll content and leaf visual score (0–9) after metribuzin application. The leaf SPAD value is linearly correlated with leaf chlorophyll concentration (Xu et al., 2020). Therefore, the leaf chlorophyll concentration was used to indicate for the level of resistance. Large differences between isolines within a pair for leaf SPAD chlorophyll content would indicate confirmed NIL pairs against metribuzin. Leaf visual score was estimated on a scale of 0 (no senescence/phytotoxicity) to 9 (senescence/dead), with a low leaf visual score indicating less chlorophyll loss and greater resistance and a high leaf visual score indicating more chlorophyll loss and greater susceptibility [8]. The greatest differences in leaf visual score occurred on the 12th day of metribuzin application.

### 4.7. Measurements of Other Morphological Traits

During the NIL assessment, other agro-morphological traits, including plant height (cm), spike length (cm), tiller number per plant, above-ground biomass (kg) per plant, TGW (g), yield (kg) per plant, powdery mildew (PM) leaf visual score, senescence leaf visual score, and physiological maturity date, were measured/counted from all putative NIL plants. Plant height (cm) was measured using a ruler just after the grain filling, starting from just above the ground to the beginning of the rachis. Days to maturity was calculated from the sowing date to 50% physiological maturity. The thousand-grain weight was obtained from counting and weighing 1000 seeds. The leaf visual scores for metribuzin application, powdery mildew (PM), and leaf senescence were estimated on a 0–9 scale [8].

### 4.8. Statistical Analysis

The visual scoring (0–9) and leaf SPAD chlorophyll content after metribuzin application, and other morphological traits obtained from each NIL pair, were analysed with a non-parametric test (Mann–Whitney *U* test) [39] using R-studio version 4.0.5.

## 5. Conclusions

The usefulness of the leaf SPAD chlorophyll content and metribuzin leaf visual score in determining metribuzin resistance was confirmed from the successful development and confirmation of seven pairs of NILs. The seven pairs of NILs were also segregated for other morphological traits, such as TGW, plant biomass per plant, tillers per plant, plant height, yield per plant, and powdery mildew (PM) visual score. TGW was found linked with metribuzin resistance in most confirmed NILs except for one pair, suggesting this 4A locus harbours closely linked genes controlling the two traits. However, multiple genes controlling other traits, including grain size, may also exist in the same region, which can be predicted from different non-overlapping MQTLs presented within the targeted QTL region. Therefore, it is necessary to characterise the locus in future studies. The confirmed contrasting metribuzin-resistant NIL pairs provide the ideal materials for transcriptomic and proteomic studies to identify candidate genes, or for fine mapping to identify the functional gene(s) for metribuzin resistance.

## Figures and Tables

**Table 1:** Plant leaf visual scores (0–9) and leaf SPAD chlorophyll contents of seven pairs of the confirmed wheat near-isogenic lines (NILs) for metribuzin resistance.

NIL Pairs as Per Figure 2	Line Name (QTL Reference: [8])	Metribuzin Leaf Visual Score (0–9)	Leaf Visual Score (0–9) Difference in Pairs (%)	SPAD	SPAD Difference in Pairs (%)
3	Qsns.uwa.4AL.3R	1.4 ± 0.071 **	80	27 ± 0.64 **	44
	Qsns.uwa.4AL.3S	7.1 ± 0.18		15 ± 1.3	
4	Qsns.uwa.4AL.4R	3.0 ± 0.28 **	61	28 ± 1.8 *	21
	Qsns.uwa.4AL.4S	7.6 ± 0.27		22 ± 0.85	
9	Qsns.uwa.4AL.9R	4.8 ± 0.18 *	47	22 ± 0.91 *	36
	Qsns.uwa.4AL.9S	9.0 ± 0.0		14 ± 1.3	
10	Qsns.uwa.4AL.10R	3.6 ± 0.17 **	48	23 ± 0.57 **	48
	Qsns.uwa.4AL.10S	6.9 ± 0.13		12 ± 0.81	
13	Qsns.uwa.4AL.13R	3.3 ± 0.25 *	56	30 ± 1.3 **	33
	Qsns.uwa.4AL.13S	7.5 ± 0.22		20 ± 1.0	
14	Qsns.uwa.4AL.14R	2.9 ± 0.37 **	60	25 ± 1.7 *	20
	Qsns.uwa.4AL.14S	7.2 ± 0.25		20 ± 0.75	
17	Qsns.uwa.4AL.17R	1.0 ± 0.0 **	86	27 ± 1.3 **	26
	Qsns.uwa.4AL.17S	7.0 ± 0.22		20 ± 1.1	

Note: Values are presented as the mean ± SE. Different lines are indicated with the QTL name followed by the line number. For example, lines Qsns.uwa.4AL.3R and Qsns.uwa.4AL.3S are two contrasting isolines of NIL pair ‘3’ targeting the 4AL QTL (Table 1). R lines are those with an allele from the resistant parent (Chuan Mai 25), and S lines are those with an allele from the susceptible parent (Ritchie). Metribuzin leaf visual scores (0–9) and leaf SPAD chlorophyll contents of seven pairs of confirmed NILs are presented. The percentage difference in leaf visual scores (0–9) are based on value differences between the isolines divided by the value of the susceptible isoline. The percentage difference in SPAD values is based on the value differences between the isolines divided by the value of the resistant isoline. ** indicates *p* ≤ 0.01 and * indicates *p* ≤ 0.05, based on Mann–Whitney *U* tests.

**Table 2:** Morphological trait differences between the isolines of each of the seven confirmed NIL pairs.

NIL Pairs	TGW (g)	Biomass (kg)/Plant	Plant Height (cm)	Tillers /Plant	Yield (g) /Plant	PM Visual Score (0–9)
3R	27 ± 0.35 **	0.45 ± 0.029	64 ± 0.33 **	4.0 ± 0.58 *	11 ± 1.0	1.0 ± 0.0
3S	23 ± 0.10	0.65 ± 0.023	59 ± 0.57	6.7 ± 0.33	12 ± 0.15	1.0 ± 0.0
4R	34 ± 0.047 **	0.55 ± 0.052	60 ± 0.33	3.7 ± 0.33	6.8 ± 2.8 **	1.0 ± 0.0 **
4S	29 ± 0.15	0.63 ± 0.0088	60 ± 0.0	4.7 ± 0.88	21 ± 1.1	7.5 ± 0.50
9R	38 ± 0.050 *	0.65 ± 0.053 *	63 ± 0.57	6.7 ± 0.88 **	32 ± 0.35 *	1.0 ± 0.0 **
9S	36 ± 0.20	0.48 ± 0.026	63 ± 1.7	3.0 ± 0.0	22 ± 0.40	7.0 ± 0.0
10R	35 ± 0.049 **	0.50 ± 0.023 **	64 ± 0.33	4.0 ± 0.58	16 ± 1.0 *	1.0 ± 0.0 **
10S	30 ± 0.30	0.29 ± 0.046	64 ± 0.57	2.0 ± 0.0	11 ± 1.2	7.5 ± 0.50
13R	40 ± 0.050 **	0.69 ± 0.039 **	65 ± 0.33	6.7 ± 0.88 *	20 ± 0.25 *	1.0 ± 0.0
13S	36 ± 0.10	0.40 ± 0.019	67 ± 0.33	3.7 ± 0.33	13 ± 0.20	1.0 ± 0.0
14R	29 ± 0.10 *	0.65 ± 0.017 **	67 ± 0.57 **	6.7 ± 0.33 **	15 ± 0.16	1.0 ± 0.0
14S	30 ± 0.20	0.26 ± 0.021	74 ± 1.1	2.0 ± 0.0	15 ± 0.85	1.0 ± 0.0
17R	38 ± 0.25 **	0.35 ± 0.0058	62 ± 1.2 **	3.0 ± 0.58	30 ± 0.078	1.0 ± 0.0
17S	27 ± 0.25	0.33 ± 0.033	69 ± 0.57	2.0 ± 0.0	32 ± 1.2	1.0 ± 0.0

Note: R lines are those with an allele from the resistant parent (Chuan Mai 25), and S lines are those with an allele from the susceptible parent (Ritchie) of the respective NIL pairs. The morphological traits of each NIL pair were tested using Mann–Whitney *U* tests. Those indicated with ** *p* ≤ 0.01 mean highly significant difference, those with * *p* ≤ 0.05 mean significant difference, and those non-significantly different pairs are not indicated. Each of the parameters was obtained from a population size (*N*) = 3. Values are presented as the mean ± SE.

## Data Availability

Not applicable.

## Supplementary Materials

The following are available online at https://www.mdpi.com/article/10.3390/plants10091856/s1, Table S1: Morphological traits of seventeen developed putative NIL pairs. Traits were presented in respective mean values ± standard errors.
