# Correction: Bhattarai et al. Characterisation of a 4A QTL for Metribuzin Resistance in Wheat by Developing Near-Isogenic Lines. *Plants* 2021, *10*, 1856

**DOI:** 10.3390/plants15101579

**Published:** 2026-05-21

**Authors:** Rudra Bhattarai, Hui Liu, Kadambot H. M. Siddique, Guijun Yan

**Affiliations:** 1 UWA School of Agriculture and Environment, The University of Western Australia, 35 Stirling Highway, Crawley, WA 6009, Australiakadambot.siddique@uwa.edu.au (K.H.M.S.); 2 The UWA Institute of Agriculture, The University of Western Australia, 35 Stirling Highway, Crawley, WA 6009, Australia

In the original publication [1], there was a mistake in Tables 1 and 2 as published. In Table 1, standard error (SE) values of the measured traits were originally shown to two decimal places but they have been corrected to two significant digits, which is the correct way to present these SE values. In addition some of the line’s Metribuzin leaf visual score mean values, leaf visual score differences in pairs (%), and SPAD differences in pairs (%) have been changed due to calculation errors found in the original manuscript. The corrected Table 1 and Table 2 are as follows:

There was a mistake in Figure 2 as published. NIL pair 18 has been removed from the data set; this removal does not impact the major findings of the article, as this pair is not a metribuzin-resistant NIL based on metribuzin assessment. The reason for removal is that one of the data values was found missing in the original dataset, which reduces the total NIL pair count. Figure 2 is revised from eighteen to seventeen NIL pairs. The corrected Figure 2 appears below.

A correction has been made to reflect the changes in numerical values in Table 1, which also applies to the manuscript abstract: “The resistant allele from the resistant parent increased metribuzin resistance by 47–86% (average 63%) compared with the susceptible allele from the susceptible parent”, result section of manuscript 2.2: “The resistant allele from the resistant parent increased metribuzin resistance by 47–86% (average 63%) compared with the susceptible allele from the susceptible parent, based on visual scoring (Table 1)”, and the manuscript paragraph 2 of result Section 2.2: “The resistant allele from the resistant parent increased metribuzin resistance by 20–48% (average 33%) compared with the susceptible allele from the susceptible parent, based on leaf SPAD chlorophyll content (Table 1).”

A correction has been made to Section 1, the second sentence of the third paragraph as follows: “Contrasting isolines are assumed to be similar in genetic backgrounds so that the trait differences between them can be attributed to the targeted gene or locus and nearby genes, facilitating the discovery of the effects of this locus or region on other traits”. The reason for this change is due to the misunderstood concept written about NIL during manuscript preparation.

The correction has been made to the seventh and eighth sentences of the fourth paragraph as follows: “Putative NILs were developed through repeated selfing using the heterogeneous inbred family (HIF) method [18] and a fast generation-cycling system (FGCS) [19]. FGCS helped to speed up the generation advancement process without losing any recombination benefit and significantly increased population development efficiency [19,20]”. The reason for this change is due to a grammatical mistake written in the sentence.

A correction has been made to the first paragraph of Section 2.1 as follows: “Seventeen heterozygous lines were identified in the F_7_ generation using the HIF method (Table S1). Two contrasting lines, each homozygous to the molecular marker alleles, were selected from the progeny of each F_7_ heterozygous plant. At F_8_, 17 putative NIL pairs were obtained and used for the metribuzin assessment. Of the 17 pairs, seven were confirmed NIL pairs against metribuzin, and others were confirmed as recombination types (Figures 1A–D and 2)”. The reason for this change is that the number of NILs changed from eighteen heterozygous lines in the original manuscript to seventeen heterozygous lines in the updated version.

A correction has been made to Section 3, the fourth sentence of the third paragraph: “As a result, only seven NILs were confirmed as true NILs”. The reason for this change is due to a change in the total number of NIL pairs, i.e., from eighteen to seventeen pairs.

A correction has been made to the fourth sentence of Section 4.5 and the third sentence of Section 4.6 as follows: “Seventeen NIL pairs were placed in seventeen such trays for the treatment and control”. “Among the seventeen putative NIL pairs, the most contrasting pairs were selected based on differences in the leaf SPAD chlorophyll content and leaf visual score (0–9) after metribuzin application”. The reason for this change is also due to the changes in the total number of NIL pairs, i.e., from eighteen pairs to seventeen pairs.

There was a mistake in the legend for Table S1. As NIL pair 18 has been removed from the dataset, reducing the total NIL pair count, a revision has been made to Table S1. The correct legend appears as follows:

Table S1: Morphological traits of seventeen developed putative NIL pairs. Traits were presented in respective mean values ± standard errors.

A correction has been made to the Table S1. The reason for this change is due to the incorrect data and the change in the SE calculation method.

The authors state that the scientific conclusions are unaffected. This correction was approved by the Academic Editor. The original publication has also been updated.

## Figures and Tables

**Figure 2:**
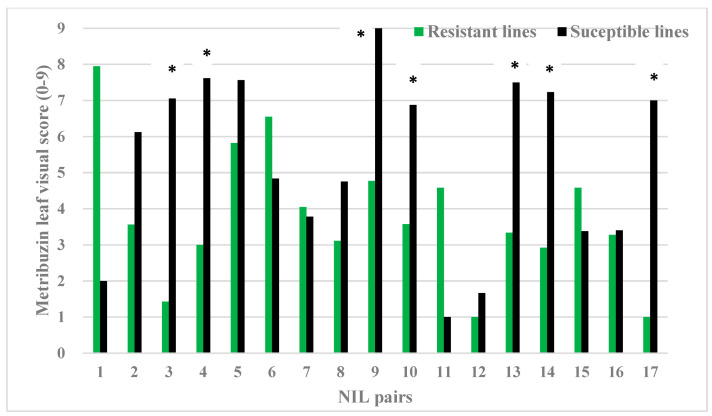
Seventeen pairs of putative near-isogenic lines (NILs) derived from F_2_ single-seed descent progenies conferring metribuzin resistance in the Chuan Mai 25 × Ritchie population were assessed with the metribuzin leaf visual score (0–9: score 0 is the most resistant and score 9 means most susceptible), which was taken on the 12th day of metribuzin application. Lines with the resistant molecular marker are green, and lines with the susceptible molecular marker are black. Confirmed NILs are marked with *.

**Table 1:** Plant leaf visual scores (0–9) and leaf SPAD chlorophyll contents of seven pairs of the confirmed wheat near-isogenic lines (NILs) for metribuzin resistance.

NIL Pairs as Per Figure 2	Line Name (QTL Reference: [8])	Metribuzin Leaf Visual Score (0–9)	Leaf Visual Score (0–9) Difference in Pairs (%)	SPAD	SPAD Difference in Pairs (%)
3	Qsns.uwa.4AL.3R	1.4 ± 0.071 **	80	27 ± 0.64 **	44
	Qsns.uwa.4AL.3S	7.1 ± 0.18		15 ± 1.3	
4	Qsns.uwa.4AL.4R	3.0 ± 0.28 **	61	28 ± 1.8 *	21
	Qsns.uwa.4AL.4S	7.6 ± 0.27		22 ± 0.85	
9	Qsns.uwa.4AL.9R	4.8 ± 0.18 *	47	22 ± 0.91 *	36
	Qsns.uwa.4AL.9S	9.0 ± 0.0		14 ± 1.3	
10	Qsns.uwa.4AL.10R	3.6 ± 0.17 **	48	23 ± 0.57 **	48
	Qsns.uwa.4AL.10S	6.9 ± 0.13		12 ± 0.81	
13	Qsns.uwa.4AL.13R	3.3 ± 0.25 *	56	30 ± 1.3 **	33
	Qsns.uwa.4AL.13S	7.5 ± 0.22		20 ± 1.0	
14	Qsns.uwa.4AL.14R	2.9 ± 0.37 **	60	25 ± 1.7 *	20
	Qsns.uwa.4AL.14S	7.2 ± 0.25		20 ± 0.75	
17	Qsns.uwa.4AL.17R	1.0 ± 0.0 **	86	27 ± 1.3 **	26
	Qsns.uwa.4AL.17S	7.0 ± 0.22		20 ± 1.1	

Note: Values are presented as the mean ± SE. Different lines are indicated with the QTL name followed by the line number. For example, lines Qsns.uwa.4AL.3R and Qsns.uwa.4AL.3S are two contrasting isolines of NIL pair ‘3’ targeting the 4AL QTL (Table 1). R lines are those with an allele from the resistant parent (Chuan Mai 25), and S lines are those with an allele from the susceptible parent (Ritchie). Metribuzin leaf visual scores (0–9) and leaf SPAD chlorophyll contents of seven pairs of confirmed NILs are presented. The percentage difference in leaf visual scores (0–9) are based on value differences between the isolines divided by the value of the susceptible isoline. The percentage difference in SPAD values is based on the value differences between the isolines divided by the value of the resistant isoline. ** indicates *p* ≤ 0.01 and * indicates *p* ≤ 0.05, based on Mann–Whitney *U* tests.

**Table 2:** Morphological trait differences between the isolines of each of the seven confirmed NIL pairs.

NIL Pairs	TGW (g)	Biomass (kg)/Plant	Plant Height (cm)	Tillers /Plant	Yield (g) /Plant	PM Visual Score (0–9)
3R	27 ± 0.35 **	0.45 ± 0.029	64 ± 0.33 **	4.0 ± 0.58 *	11 ± 1.0	1.0 ± 0.0
3S	23 ± 0.10	0.65 ± 0.023	59 ± 0.57	6.7 ± 0.33	12 ± 0.15	1.0 ± 0.0
4R	34 ± 0.047 **	0.55 ± 0.052	60 ± 0.33	3.7 ± 0.33	6.8 ± 2.8 **	1.0 ± 0.0 **
4S	29 ± 0.15	0.63 ± 0.0088	60 ± 0.0	4.7 ± 0.88	21 ± 1.1	7.5 ± 0.50
9R	38 ± 0.050 *	0.65 ± 0.053 *	63 ± 0.57	6.7 ± 0.88 **	32 ± 0.35 *	1.0 ± 0.0 **
9S	36 ± 0.20	0.48 ± 0.026	63 ± 1.7	3.0 ± 0.0	22 ± 0.40	7.0 ± 0.0
10R	35 ± 0.049 **	0.50 ± 0.023 **	64 ± 0.33	4.0 ± 0.58	16 ± 1.0 *	1.0 ± 0.0 **
10S	30 ± 0.30	0.29 ± 0.046	64 ± 0.57	2.0 ± 0.0	11 ± 1.2	7.5 ± 0.50
13R	40 ± 0.050 **	0.69 ± 0.039 **	65 ± 0.33	6.7 ± 0.88 *	20 ± 0.25 *	1.0 ± 0.0
13S	36 ± 0.10	0.40 ± 0.019	67 ± 0.33	3.7 ± 0.33	13 ± 0.20	1.0 ± 0.0
14R	29 ± 0.10 *	0.65 ± 0.017 **	67 ± 0.57 **	6.7 ± 0.33 **	15 ± 0.16	1.0 ± 0.0
14S	30 ± 0.20	0.26 ± 0.021	74 ± 1.1	2.0 ± 0.0	15 ± 0.85	1.0 ± 0.0
17R	38 ± 0.25 **	0.35 ± 0.0058	62 ± 1.2 **	3.0 ± 0.58	30 ± 0.078	1.0 ± 0.0
17S	27 ± 0.25	0.33 ± 0.033	69 ± 0.57	2.0 ± 0.0	32 ± 1.2	1.0 ± 0.0

Note: R lines are those with an allele from the resistant parent (Chuan Mai 25), and S lines are those with an allele from the susceptible parent (Ritchie) of the respective NIL pairs. The morphological traits of each NIL pair were tested using Mann–Whitney *U* tests. Those indicated with ** *p* ≤ 0.01 mean highly significant difference, those with * *p* ≤ 0.05 mean significant difference, and those non-significantly different pairs are not indicated. Each of the parameters was obtained from a population size (*N*) = 3. Values are presented as the mean ± SE.
